# Psychological Impact of Long‐Term Dementia Caregiving: A Study on Family Caregivers in Taiwan

**DOI:** 10.1002/alz.095360

**Published:** 2025-01-09

**Authors:** HAN‐KUAN BAI, CHINGCHING LU, Fang Liu

**Affiliations:** ^1^ Taipei Medical University, Taipei City, Taiwan Taiwan; ^2^ National Tsing Hua University, Hsinchu City, Taiwan Taiwan

## Abstract

**Background:**

As global populations age, dementia prevalence is increasing, with projections suggesting significant growth in the number of affected individuals and their caregivers. In Taiwan, family caregivers provide substantial support, often facing intense burdens due to prolonged caregiving duties. This study aims to assess the psychological health of these caregivers to better understand and address their needs.

**Method:**

This research involved 20 long‐term family caregivers of dementia patients. We used several psychometric instruments to assess their psychological status: the Pittsburgh Sleep Quality Index (PSQI), the Perceived Stress Scale (PSS), and Beck’s Depression Inventory (BDI). Measurements were taken twice, one month apart, to ensure consistency over time.

**Result:**

The findings indicate severe psychological impacts among caregivers. Ninety percent of participants reported chronic insomnia as indicated by a PSQI score above 5. The PSS results showed that 65% of caregivers experienced abnormal stress levels. Depression was prevalent, with 60% of caregivers showing depressive symptoms; 30% were mildly depressed, 20% moderately, and 10% severely.

**Conclusion:**

The study highlights the critical psychological toll on family members providing long‐term care to dementia patients. The severity of mental health issues among these caregivers underscores the urgent need for comprehensive support systems that can alleviate their burden and improve their quality of life.

